# Early therapy contributes to the normalization of platelet in patients with severe fever with thrombocytopenia syndrome during the convalescent phase

**DOI:** 10.1371/journal.pntd.0012793

**Published:** 2025-01-13

**Authors:** Xiaoyu Xue, Xiaolei Wang, Ling Lin, Wenjing Niu, Zhouling Jiang, Kehang Liu, Yanli Xu, Youde Liu, Zhihai Chen

**Affiliations:** 1 Department of Infectious Disease, Peking University Ditan Teaching Hospital, Beijing, China; 2 The 2nd Affiliated Hospital of Harbin Medical University, Harbin Medical University, Harbin, Heilongjiang, China; 3 Department of Infectious Diseases, Yantai Qishan Hospital, Yantai, China; 4 National Key Laboratory of Intelligent Tracking and Forecasting for Infectious Diseases, Beijing Ditan Hospital, Capital Medical University, Beijing, China; Instituto Butantan, BRAZIL

## Abstract

**Background:**

Platelet recovery was an important prognostic indicator in severe fever with thrombocytopenia syndrome (SFTS). This study focused on risk factors affecting platelet recovery in surviving SFTS patients, which can assist clinicians in the early screening of patients associated with a greater risk of mortality.

**Method:**

We retrospectively analyzed the clinical data of SFTS patients admitted to Yantai Qishan Hospital throughout 2023. According to the Diagnosis and Treatment Guideline (2023 edition), the platelet recovery in 14 days was set as outcome. The multivariate Cox regression was used to identify independent risk factors affecting platelet recovery and the Kaplan-Meier was performed to evaluate the probability of 14-day platelet recovery, using receiver operating characteristic (ROC) curve and area under the curve (AUC) to measure the model’s performance, with clinical benefit assessed by decision curve analysis (DCA).

**Results:**

168 SFTS patients were enrolled in the study, with 76.2% (128/168) achieving platelet (PLT) recovery within 14 days. Independent risk factors were baseline PLT > 90 × 10^9^/L (HR: 7.929, 95%CI: 1.066–58.990, *P* = 0.043), days from onset to admission >6 days (HR: 0.444, 95%CI: 0.259–0.763, *P* = 0.003) and baseline prothrombin time (PT) >13 s (HR: 0.547, 95%CI: 0.373–0.800, *P* = 0.002), with an AUC of 0.745 (95% CI: 0.656–0.834, *P* < 0.001). DCA demonstrated that when the recovery probability beyond approximately 50%, the clinical net benefit from focusing on the PLT stratification model consistently surpassed that from the all-intervention model. The nomogram further visualized the model.

**Conclusion:**

Early diagnosis and timely therapy contributed to recovery during convalescence in SFTS patients, with baseline PT as a strong predictor.

## Author summary

Previous studies have mainly focused on the risk factors of death in SFTS patients. The mortality rate of SFTS in China has decreased from 10.58% to 5.07% over the past decade, therefore we pay more attention to the prognosis of SFTS patients who survive during convalescence. The recovery of PLT level during convalescence is one of the important indicators for measuring patient improvement, thus our study analyzed the predictors influencing PLT recovery during convalescence. In this study, we found that baseline PT, days from onset to admission, and PLT at baseline were independent risk factors affecting PLT recovery, which emphasized the importance of early diagnosis and timely treatment in SFTS patients. Our study provides evidence that early diagnosis and timely treatment contribute to improving the prognosis of SFTS patients, which can assist clinicians in the early screening of patients associated with a greater risk of adverse sequela and mortality in the future.

## Introduction

Severe fever with thrombocytopenia syndrome (SFTS) is an acute infectious disease caused by a novel bunyavirus known as SFTS virus (SFTSV) primarily transmitted by tick bite and occasionally from person-to-person spread via SFTSV-infected blood or fluid [1]. It is characterized mainly by fever, thrombocytopenia, and leukopenia, whereas SFTSV infected directly the central nervous system in severe cases, leading to SFTS-associated encephalopathy/encephalitis (SFTSAE) [2]. Thrombocytopenia (PLT < 100 × 10^9^/L) is a consistent feature of SFTSV infection, which may be attributed to the two potential mechanisms: the binding of SFTSV with platelet glycoprotein VI, leading to exposure of phosphatidylserine on the platelet surface and further inducing phagocytosis of platelets by macrophages in the spleen [3]; and platelet arginine deficiency and subsequent decreased intraplatelet nitric oxide (Plt-NO) might contribute to the platelet activation, which was possibly associated with platelet-monocyte aggregation and platelet apoptosis [4]. There are currently no specific antiviral drugs or vaccines for SFTS, and the mortality rate has been as high as 30 percent in previous cases of SFTS [5]. Therefore, many studies have established mortality risk prediction models, which have shown that age, longer onset-to-hospitalization time, prolonged prothrombin time, and PLT < 20 × 10^9^/L were independent risk factors for SFTS mortality [6–8]. Recently, with the deeper understanding of SFTSV pathogenesis and improvement of the treatment, the mortality rate of SFTS in China has obviously decreased [9]. Therefore, the clinician focused more on the prognosis of surviving SFTS patients during convalescence.

According to the clinical occurrence, progression and serological changes of SFTS, the typical course of infection has four distinct periods: incubation, fever, multiple organ failure, and convalescence. A previous study revealed that the convalescence period for surviving patients typically ranges from 11 to 19 days after onset [10], in addition, the SFTS Diagnosis and Treatment Guideline (2023 edition) suggests that it usually resolves about two weeks (14 days) into the disease course, and may be prolonged in the presence of complications [1]. The recovery of PLT level during convalescence is one of the important indicators for measuring patient improvement [11]. However, few researches that illustrated the factors influencing PLT recovery (≥100 × 10^9^/L) during convalescence of SFTS patients. Therefore, our study aimed to identify associated risk factors affecting timely PLT recovery in most patients during convalescence, which can assist clinicians earlier in recognizing and facilitates to carrying out of clinical intervention, thereby enhancing patient prognosis.

## Method

### Ethics statement

This study was conducted according to the principles of the Helsinki Declaration and was approved by the Human Science and Ethics Committee of Beijing Ditan Hospital, Capital Medical University (no. DTEC-KY2022-022-01). All clinical and laboratory data were used anonymously, and informed consent was waived due to the retrospective study.

### Research design

**Fig 1:**
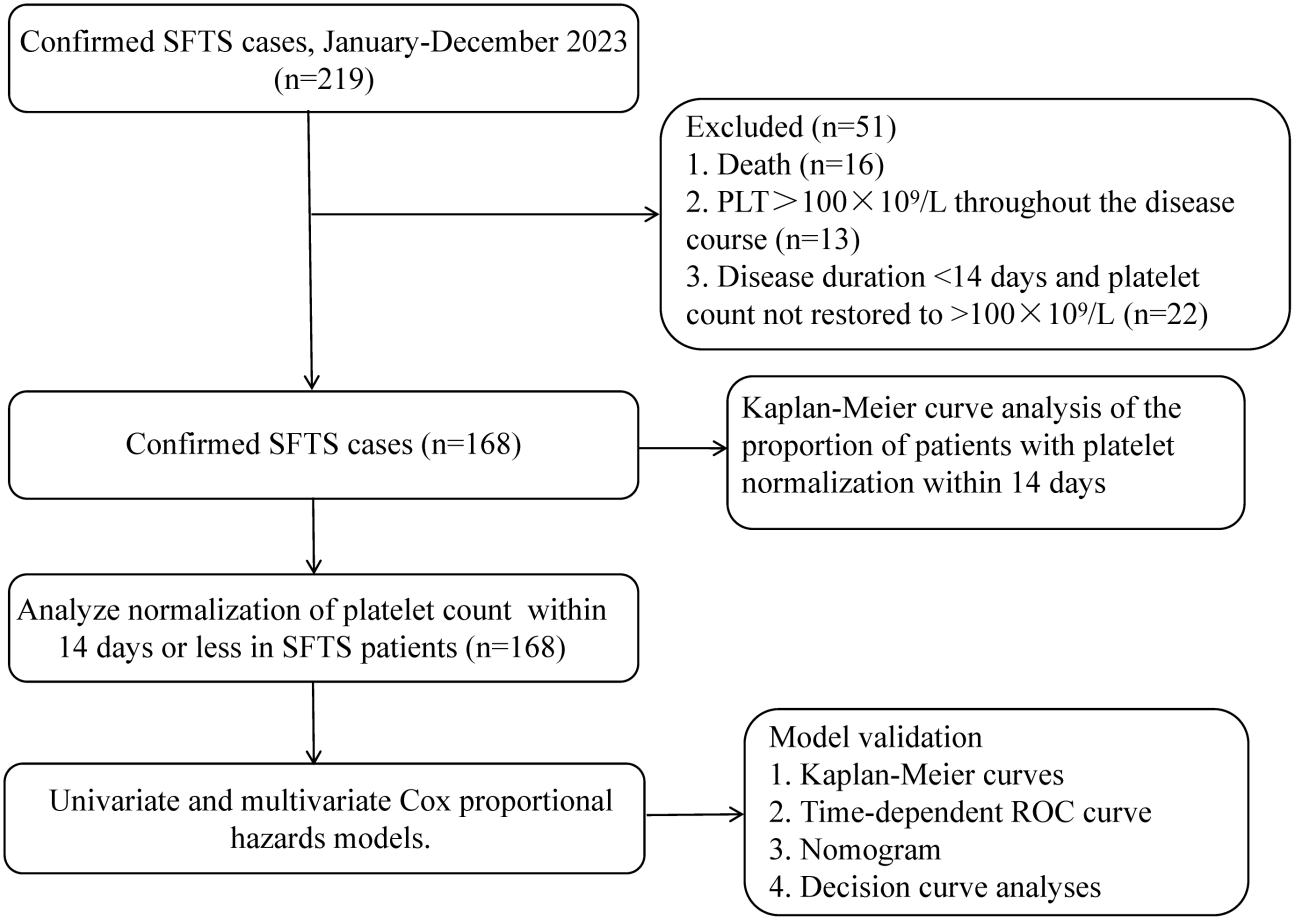
Flowchart of SFTS patients for inclusion in study analysis. Abbreviations: SFTS: severe fever with thrombocytopenia syndrome, ROC: receiver operating characteristic, PLT: platelet.

The study data were collected from 219 confirmed SFTS patients from January to December 2023 at Yantai Qishan Hospital in Shandong Province. Participants were included based on the following inclusion criteria [1]: 1. Epidemiological history (a record of working, living, or traveling in hilly, forested, or mountainous areas during the epidemic season, as well as a history of tick bites or contact with SFTS patients within 2 weeks before the onset of the disease), 2. Clinical manifestations included fever and/or bleeding, accompanied by decreased platelet and white blood cell counts in peripheral blood, 3. Diagnosis could be confirmed if any of the following were met: (1) Positive plasma SFTSV nucleic acid test (real-time fluorescent polymerase chain reaction, RT-PCR); (2) Positive plasma SFTS IgM antibody test; (3) SFTS IgG antibodies turned positive or showed a fourfold or greater increase in titer during convalescence compared to the acute phase. The exclusion criteria were the following: 1. death (n = 16), 2. PLT >100 × 10^9^/L by laboratory tests throughout the duration of disease (n = 13), 3. The duration of the disease was less than 14 days and PLT did not recover to >100 × 10^9^/L (n = 22). Based on the above inclusion and exclusion criteria, 168 patients with SFTS were finally included in this study (Fig 1).

### Data collection

The demographic data, epidemiological data, baseline clinical presentation, and baseline laboratory indicators were collected for each patient from medical record, including age, gender, days from onset to admission, length of hospital stay, duration of disease, history of tick bites, fever, highest body temperature, cough, diarrhea, neurological signs, routine blood tests (white blood cell count [WBC], platelet [PLT], hemoglobin [HGB], lymphocyte count), coagulation function (prothrombin time [PT]), myocardial enzymes (creatine kinase [CK]), and electrolytes (calcium ions [Ca^2+^]). In addition, treatment information collected from cases included the application of antivirals (favipiravir/ribavirin), symptomatic and supportive therapies (antibacterial therapy, antifungal therapy, corticosteroid therapy, recombinant human granulocyte colony-stimulating factor (rhG-CSF) therapy, intravenous immunoglobulin (IVIG), continuous renal replacement therapy (CRRT), platelet transfusions [12]. Comorbidities in our study referred to those that associated with the progression of SFTS, including lung involvement (Mechanical ventilation), acute kidney injury, bacterial pneumonia, fungal pneumonia [13].

### Clinical definition

The PLT recovery was considered as the primary positive outcome and defined as a value ≥ 100 × 10^9^/L. The days from onset to admission was divided using 3 days and 6 days as cutoff points [14]. Body temperature was categorized into low-grade fever (37.5–38 °C), moderate fever (38.1–39 °C), high fever (39.1–40 °C), and hyperpyrexia (>40 °C). Lymphocyte count was divided using 2 × 10^9^/L as a cutoff point [15], while prothrombin time >13 s, creatine kinase >200 U/L (lower limit of normal), and calcium ion >2.2 mmol/L (upper limit of normal) were used as cut-off points [16]. Neurological signs were defined as the presence of at least one of the following changes: muscle tension, involuntary movements, or abnormal reflexes. Acute kidney injury referred to as an increase in serum creatinine of ≥0.3 mg/dL within 48 hours [17]. Bacterial pneumonia was defined as the presence of one of the following bacterial infections: *Escherichia coli*, *Klebsiella pnenmoniae*, *Pseudomonas aeruginosa*, *Acinetobacter baumannii*, *Haemophilus influenzae*, *streptococcus*. Fungal pneumonia was defined as the presence of *aspergillus* or *Candida albicans*. Sensitive antibiotics and antifungals should be selected based on above infections. Platelet transfusions for significant bleeding or low platelet count (<30 × 10^9^/L), and rhG-CSF for severe neutrophil depression (<1 × 10^9^/L) [18]. The treatment was classified into three categories: antivirals (any one), symptomatic and supportive therapies (any one or more), and combination therapy, which was referred to the simultaneous administration of an antiviral drug and one or more symptomatic therapies.

### Statistical analysis

The sample size was estimated by using PASS 15.0 software. Assuming a 89.5% difference in survival rates as has been demonstrated in the previous study [19], with a significance level of 0.05 (two-sided). The estimated sample size was 164, which was almost consistent with our study. The quantitative data following normal distribution were expressed as mean ± standard deviation and analyzed using the independent samples t-tests. For quantitative data not conforming to normal distribution, the median (M) and interquartile range (IQR) were utilized, alongside the Mann-Whitney U tests for comparative analysis. Qualitative data were presented as numbers and percentages, which were evaluated with the chi-squared tests, continuity correction, or Fisher’s exact probability tests. Variables with more than 30% missing values were excluded from statistical analysis. For variables with 30% or less missing values, missing values were replaced with the mean for normally distributed data and the median for non-normally distributed data.

Referring to clinical practice and previous reports, continuous variables (age, days from onset to admission, PLT, Lymphocyte count, PT, CK, Ca^2+^, treatment) were converted into categorical variables for Cox proportional hazard models. Predictors of 14-day PLT recovery that showed *P* < 0.1 in univariate analysis were included in the subsequent multivariate Cox regression using the forward stepwise selection process (Likelihood Ratio) method to evaluate independent risk factors. The probabilities of PLT recovery were estimated using the Kaplan-Meier method and compared using log-rank tests based on the categories of independent risk factors selected from the multivariate Cox regression. The Time-dependent ROC curve was utilized to assess the predictive power of the model, which was performed by Hiplot Pro (https://hiplot.com.cn/), an integrated web service for biomedical data analysis and visualization. Finally, the nomogram model was created utilizing *RMS* in the R package (version 4.3.3; R studio, Boston, Massachusetts, USA) to make the outcomes more visualized, and the decision curve analysis (DCA) curve was performed using *ggDCA* in the R package to measure the clinical net benefit of the Cox model. Statistical analysis was performed using SPSS software (version 25.0, IBM, Armonk, NY, USA), GraphPad (version 9.0, GraphPad Software, San Diego, California), and R studio (version 4.3.3; Boston, Massachusetts, USA). All tests were two-tailed, and the significance was set at 0.05.

## Results

### Baseline characteristics

A total of 168 patients were enrolled in the study, comprising 128 in the recovery group and 40 in the non-recovery group. Of these, 70 were male and 98 were female, with a median age of 65 years (IQR, 58–72). The average age was 63.1 years (63.1 ± 11.4) in the recovery group and 67.3 years (67.3 ± 7.8) in the non-recovery group. There was no statistically significant difference in gender between the two groups (*P* = 0.391). Significant differences were observed between the two groups in terms of the stratification of days from onset to admission, length of hospital stay, and duration of disease (*P* < 0.001), with the non-recovery group experiencing longer hospital stays and duration of disease. The non-recovery group had a higher incidence of diarrhea (*P* = 0.007), while no significant differences were noted in symptoms of fever, phlegm, and neurological signs (*P* > 0.05). The baseline PLT of recovery group [74.0 (61.3–86.8)] was higher than non-recovery group (59.0 ± 18.8) (*P* < 0.001). The baseline stratification of PLT and PT showed significant differences between the two groups (*P* < 0.05). The non-recovery group was associated with a disproportionately higher incidence of the need for mechanical ventilation (25%), bacterial pneumonia (40%), and fungal pneumonia (35%) during the hospitalization course (*P* < 0.05). The non-recovery group had a higher application proportion of antifungal therapy (50%), IVIG (30%), and CRRT (10%) (*P* < 0.05). There were a total of 8 patients received platelet transfusions without significant differences between the two groups [4 (3.1) vs 4 (10.0)] (*P* = 0.175) (Table 1).

**Table 1:** Baseline characteristics of SFTS patients in both groups (n = 168).

Characteristics	Total (n = 168)	Achieved PLT ≥ 100 × 10^9^/L (n = 128)	Not Achieved PLT ≥ 100 × 10^9^/L (n = 40)	Statistics (χ2/Z/t)	*P*-value
Age (years)	65.0 (58.0–72.0)	63.1 ± 11.4	67.3 ± 7.8	2.610	0.011
Sex (male)	70 (41.7)	51 (39.8)	19 (47.5)	0.735	0.391
Days from onset to admission (day)				8.044	0.018
≤3	25 (14.9)	20 (15.6)	5 (12.5)		
3–6	74 (44.0)	63 (49.2)	11 (27.5)		
>6	69 (41.1)	45 (35.2)	24 (60.0)		
Duration of hospitalisation (day)	9.0 (8.0–12.0)	9.0 (7.0–11.0)	12.0 (10.0–15.0)	−4.958	<0.001
Course of disease (day)	15.0 (13.0–18.0)	14.0 (13.0–17.0)	19.0 (17.0–23.8)	−6.622	<0.001
History of tick bites				0.636	0.728
Yes	47 (28.0)	34 (26.6)	13 (32.5)		
No	39 (23.2)	31 (24.2)	8 (20.0)		
Unknown	82 (48.8)	63 (49.2)	19 (47.5)		
**Clinical Manifestation**					
Fever	161 (95.8)	123 (96.1)	38 (95)	0.000	1.000
Highest temperature (°C)				3.037	0.436
37.5–38	26 (16.6)	21 (17.5)	5 (13.5)		
38.1–39	98 (62.4)	75 (62.5)	23 (62.2)		
39.1–40	32 (20.4)	24 (20.0)	8 (21.6)		
>40	1 (0.6)	0	1 (2.7)		
Phlegm	18 (10.7)	10 (7.8)	8 (20.0)	3.544	0.060
Diarrhea	70 (41.7)	46 (35.9)	24 (60.0)	7.260	0.007
Neurological signs	45 (26.8)	30 (23.4)	15 (37.5)	3.073	0.080
**Laboratory Parameters on Admission**					
White blood cells (×10^9^/L)	2.6 (1.6–3.8)	2.7 (1.7–4.2)	2.1 (1.3–3.4)	−1.762	0.078
Platelet counts (×10^9^/L)	71.0 (56.3–83.0)	74.0 (61.3–86.8)	59.0 ± 18.8	−3.906	<0.001
				16.128	0.001
≤30	5 (3.0)	1 (0.8)	4 (10.0)		
30–60	46 (27.4)	29 (22.7)	17 (42.5)		
60–90	92 (54.8)	75 (58.6)	17 (42.5)		
>90	25 (14.9)	23 (18.0)	2 (5.0)		
Hemoglobin (g/L)	142.4 ± 16.8	141.8 ± 16.4	144.3 ± 18.0	0.822	0.412
Lymphocytes (×10^9^/L)				0.542	0.462
<2	163 (97.0)	123 (96.1)	40 (100)		
≥2	5 (3.0)	5 (3.9)	0		
PT (s)				5.415	0.020
≤13	98 (58.3)	81 (63.3)	17 (42.5)		
>13	70 (41.7)	47 (36.7)	23 (57.5)		
CK (U/L)				0.100	0.752
<200	43 (25.6)	32 (25.0)	11 (27.5)		
≥200	125 (74.4)	96 (75.0)	29 (72.5)		
Ca^2+^ (mmol/L)				0.542	0.462
≤2.2	163 (97.0)	123 (96.1)	40 (100)		
2.2–2.55	5 (3.0)	5 (3.9)	0		
Comorbidity					
Lung involvement (Mechanical ventilation)	18 (10.7)	8 (6.3)	10 (25.0)	9.326	0.002
Acute kidney injury	15 (8.9)	9 (7.0)	6 (15.0)	1.501	0.221
Bacterial pneumonia	31 (18.5)	15 (11.7)	16 (40.0)	16.199	<0.001
Fungal pneumonia	31 (18.5)	17 (13.3)	14 (35.0)	9.554	0.002
Treatment					
Antivirals					
Famviravir	155 (92.3)	116 (90.6)	39 (97.5)	1.170	0.279
Ribavirin	17 (10.1)	15 (11.7)	2 (5.0)	0.864	0.353
Symptomatic and supportive therapies					
Antibacterial therapy	76 (45.2)	56 (43.8)	20 (50.0)	0.481	0.488
Antifungal therapy	48 (28.6)	28 (21.9)	20 (50.0)	11.813	0.001
Corticosteroid therapy	85 (50.6)	62 (48.4)	23 (57.5)	1.001	0.317
rhG-CSF therapy	81 (48.2)	58 (45.3)	23 (57.5)	1.813	0.178
IVIG	29 (17.3)	17 (13.3)	12 (30.0)	5.965	0.015
CRRT	5 (3.0)	1 (0.8)	4 (10.0)	6.061	0.014
Platelet transfusions	8 (4.8)	4 (3.1)	4 (10.0)	1.841	0.175

Descriptive data are n (%), median (IQR), or mean ± standard deviation. Days from onset to admission: range of 3–6 days includes 6 days; platelet stratification: range of 30–60 includes 60 and range of 60–90 includes 90; calcium stratification: range of 2.2–2.55 includes 2.55. Abbreviations: PT: Prothrombin time, CK: Creatine phosphokinase, Ca^2+^: calcium, SFTS: Severe fever with thrombocytopenia syndrome, CRRT, continuous renal replacement therapy, IVIG: intravenous immunoglobulin, rhG-CSF: recombinant human granulocyte colony-stimulating factor.

### Determinants of PLT recovery

Univariable Cox regression analysis of the PLT recovery within 14 days identified several factors: stratification of days from onset to admission, baseline stratification of PLT, lymphocytes, PT, age, and Ca^2+^, as well as phlegm, Neurological signs, and diarrhea (*P* < 0.1). These variables were subsequently included in a multivariate analysis, which determined three independent predictors of PLT recovery: stratification of days from onset to admission, baseline PT, and baseline PLT (*P* < 0.05). Days from onset to admission greater than 6 days (HR: 0.444, 95%CI: 0.259–0.763, *P* = 0.003) and PT greater than 13s (HR: 0.547, 95%CI: 0.373–0.800, *P* = 0.002) were identified as risk factors for PLT recovery, and baseline PLT greater than 90 × 10^9^/L (HR: 7.929, 95%CI: 1.066–58.990, *P* = 0.043) contributed to PLT recovery (Table 2). A predictive equation was established via Cox regression analysis: PLT recovery index = (1.297/1.827/2.156) × PLT−0.672×PT+(−0.104/−0.856) × days from onset to admission. Scores were assigned as follows: 0 for patients with PLT ≤ 30 × 10^9^/L, PT ≤ 13s, and days from onset to admission ≤3 days; 1 for patients with days from onset to admission between 3 and 6 days, PLT between 30 and 60 × 10^9^/L, and PT > 13 s; 2 for patients with days from onset to admission >6 days, and PLT between 60 and 90 × 10^9^/L; 3 for patients with PLT > 90 × 10^9^/L.

**Table 2:** Cox proportional hazard regression analysis to identify factors associated with PLT ≥ 100 × 10^9^/L.

Variables	Univariate			Wald χ^2^ (Excluded)	*P*-value (Excluded)	Multivariate		
	HR	95%CI	*P*-value			HR	95%CI	*P*-value
Age (years)				1.981	0.159	Not included		
≤60	Ref							
>60	0.686	(0.482–0.974)	0.035					
Sex								
Male	Ref							
Female	1.126	(0.790–1.604)	0.511					
Days from onset to admission (day)								
≤3	Ref					Ref		
3–6	0.83	(0.502–1.373)	0.468			0.876	(0.528–1.452)	0.608
>6	0.484	(0.285–0.821)	0.007			0.444	(0.259–0.763)	**0.003**
Highest temperature (°C)								
37.5–38	Ref							
38.1–39	0.862	(0.531–1.400)	0.549					
39.1–40	0.791	(0.440–1.422)	0.433					
>40	0	(0–1.252E+179)	0.959					
Fever								
Negative	Ref							
Positive	1.084	(0.443–2.652)	0.859					
Phlegm				1.208	0.272	Not included		
Negative	Ref							
Positive	0.538	(0.282–1.027)	0.06					
Diarrhea				2.600	0.107	Not included		
Negative	Ref							
Positive	0.668	(0.465–0.960)	0.029					
Neurological signs				2.078	0.149	Not included		
Negative	Ref							
Positive	0.676	(0.449–1.018)	0.061					
White blood cells (×10^9^/L)	1.035	(0.968–1.105)	0.315					
Platelet counts (×10^9^/L)								
≤30	Ref					Ref		
30–60	4.042	(0.551–29.684)	0.17			3.512	(0.478–25.813)	0.217
60–90	6.969	(0.968–50.175)	0.054			5.75	(0.797–41.507)	0.083
>90	10.584	(1.427–78.496)	0.021			7.929	(1.066–58.990)	**0.043**
Hemoglobin (g/L)	0.997	(0.987–1.007)	0.531					
Lymphocytes (×10^9^/L)				2.255	0.133	Not included		
<2	Ref							
≥2	2.176	(0.884–5.356)	0.091					
PT (s)								
≤13	Ref					Ref		
>13	0.636	(0.444–0.913)	0.014			0.547	(0.373–0.800)	**0.002**
CK (U/L)								
<200	Ref							
≥200	1.095	(0.734–1.634)	0.657					
Ca^2+^ (mmol/L)				1.843	0.175	Not included		
≤2.2	Ref							
2.2–2.55	2.627	(1.070–6.446)	0.035					
Treatment								
Antivirals	Ref							
Symptomatic and supportive therapies	1.399	(0.534–3.663)	0.494					
Combination therapy	0.762	(0.491–1.183)	0.227					

Days from onset to admission: range of 3–6 days includes 6 days; platelet stratification: range of 30–60 includes 60 and range of 60–90 includes 90; calcium stratification: range of 2.2–2.55 includes 2.55. Abbreviations: PT: Prothrombin time, CK: Creatine phosphokinase, Ca^2+^: calcium, HR: Hazard ratio, CI: Confidence interval.

Wald χ² scores represent the contribution of excluded variables during the forward stepwise selection process (Likelihood Ratio). Not included: variables not included in the multivariate model were excluded due to non-significance (*P* > 0.05) or multicollinearity.

### Validation of the PLT recovery model

**Fig 2:**
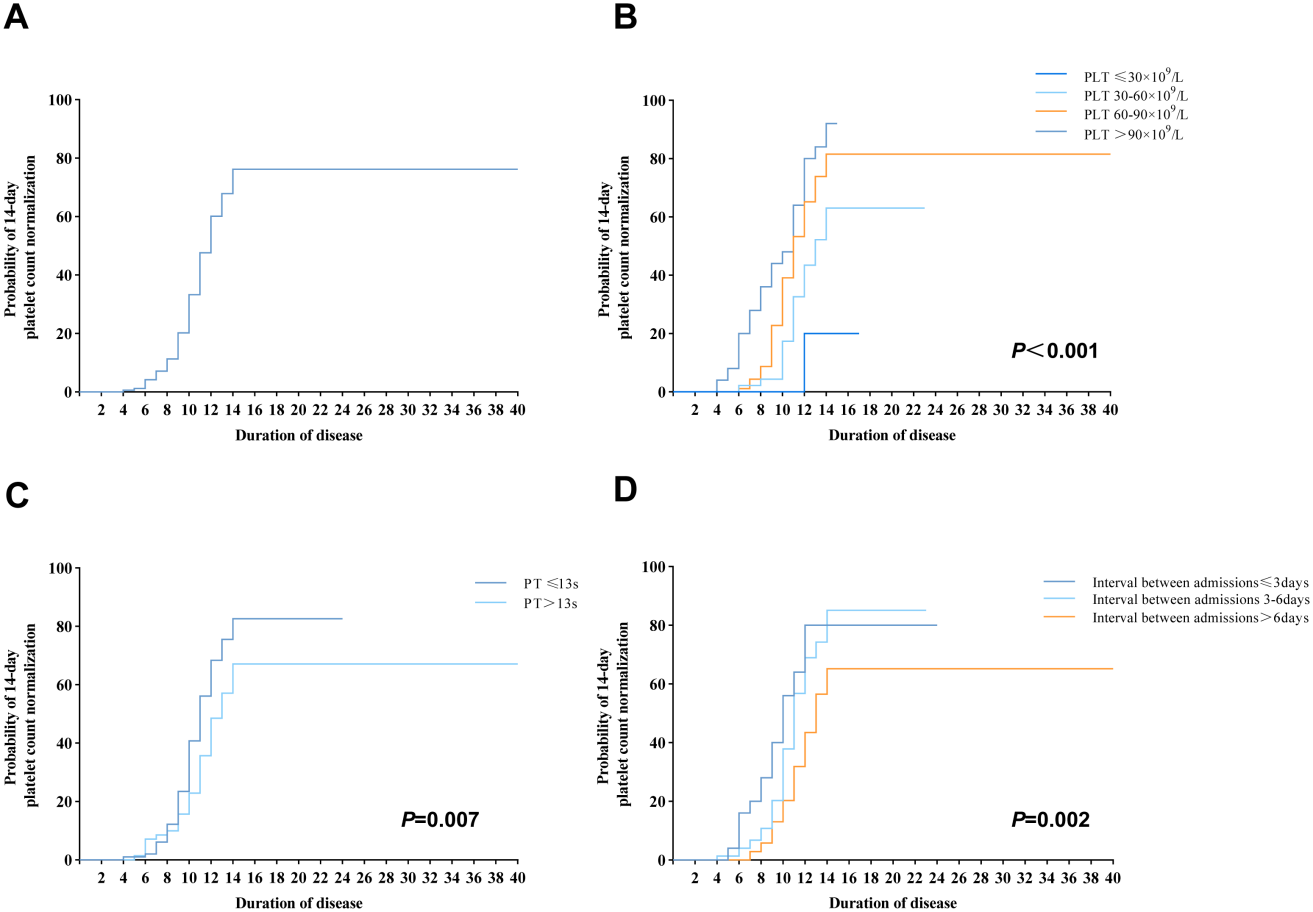
Probability of PLT recovery in SFTS patients. (A) The probability of PLT recovery estimated by the Kapla-Meier method. (B) Probability of PLT recovery by baseline platelet count. (C) Probability of PLT recovery by PT at baseline. (D) Probability of PLT recovery by the days from onset to admission. Abbreviations: PLT: platelet, PT: prothrombin time. SFTS: severe fever with thrombocytopenia syndrome.

**Fig 3:**
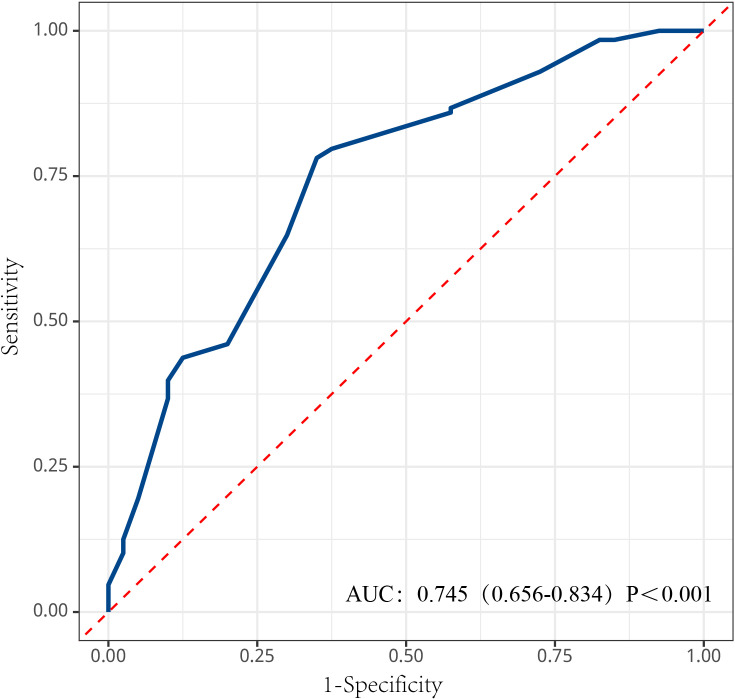
Time-dependent receiver operating characteristic (ROC) curve of the Cox regression model for predicting the likelihood of PLT recovery within 14 days in SFTS patients, which led to a diagnostic yield of 74.5% measured by the area under the ROC curve (AUC) (95% CI,0.656–0.834, *P* < 0.001). Abbreviations: CI: confidence interval, AUC: area under the curve, SFTS: severe fever with thrombocytopenia syndrome, ROC: receiver operating characteristic, PLT: platelet.

The probabilities of PLT recovery within 14 days as time to onset increased were estimated using the Kaplan-Meier curves, and resultantly, the probabilities at 7 days, 8 days, 10 days, 12 days, and 14 days were determined to be 7.1%, 11.3%, 33.3%, 60.1%, and 76.2%, respectively. It can be seen that recovery was slowest during the first 6 days of illness and increased rapidly from 7 to 14 days, with most patients achieving a normal platelet count within 14 days (Fig 2A). The probability of PLT recovery, which varied according to baseline PLT count (Fig 2B); patients with a PLT count of less than or equal to 30 × 10^9^/L at baseline had a 14-day probability of achieving a PLT recovery of 20%, compared with 63.0% among those with a PLT count at baseline between 30 × 10^9^/L and 60 × 10^9^/L, 81.0% among those with a baseline PLT count between 60 × 10^9^/L and 90 × 10^9^/L, and 92.0% among those with a PLT count at baseline more than 90 × 10^9^/L (*P* < 0.001). The probability of PLT recovery within 14 days, based on the stratification of PT at baseline, with 82% for patients with a baseline PT ≤ 13s and 67% for those with a baseline PT > 13s (*P* = 0.007) (Fig 2C). According to the stratification by days from onset to admission, patients admitted within 3 days had a 14-day recovery probability of 80%, compared to 85% for those admitted between 3 and 6 days, and 65% for those admitted after more than 6 days (*P* = 0.002) (Fig 2D). The time-dependent ROC curve was used to assess the predictive ability of the Cox regression model, which achieved an AUC of 0.745 (95%CI: 0.656–0.834, *P* < 0.001), indicating excellent predictive value (Fig 3).

### Visualization and assessment clinical benefit of the PLT recovery model

**Fig 4:**
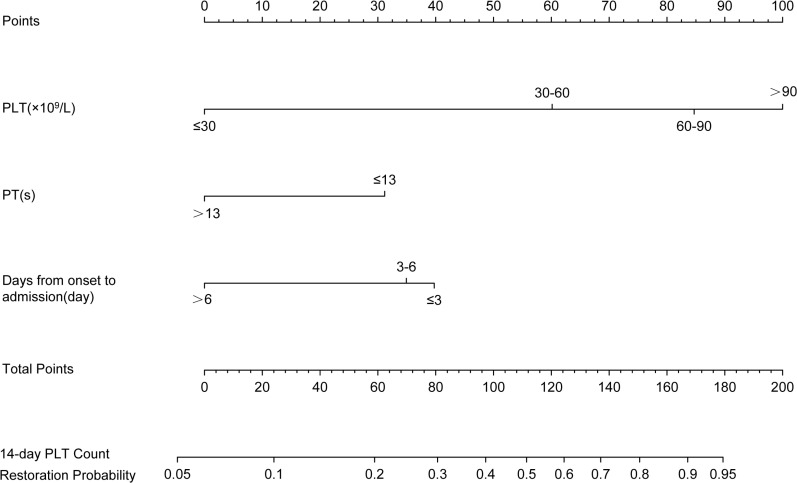
The nomogram predicted the PLT recovery rate of SFTS patients in the 14-day. To use the nomogram, the value of an individual patient is located on each variable axis, and a line is drawn upward to determine the number of points received for the value of each variable. The sum of these numbers is located on the total point axis, and a line is drawn downward to the 14-day axes to determine the likelihood of PLT recovery. 0 points were assigned for PLT ≤ 30 × 10^9^/L, PT > 13s, and days from onset to admission > 6 days, and 100 points were assigned for PLT > 90 × 10^9^/L. Abbreviations: PLT: platelet, PT: prothrombin time, SFTS: severe fever with thrombocytopenia syndrome.

**Fig 5:**
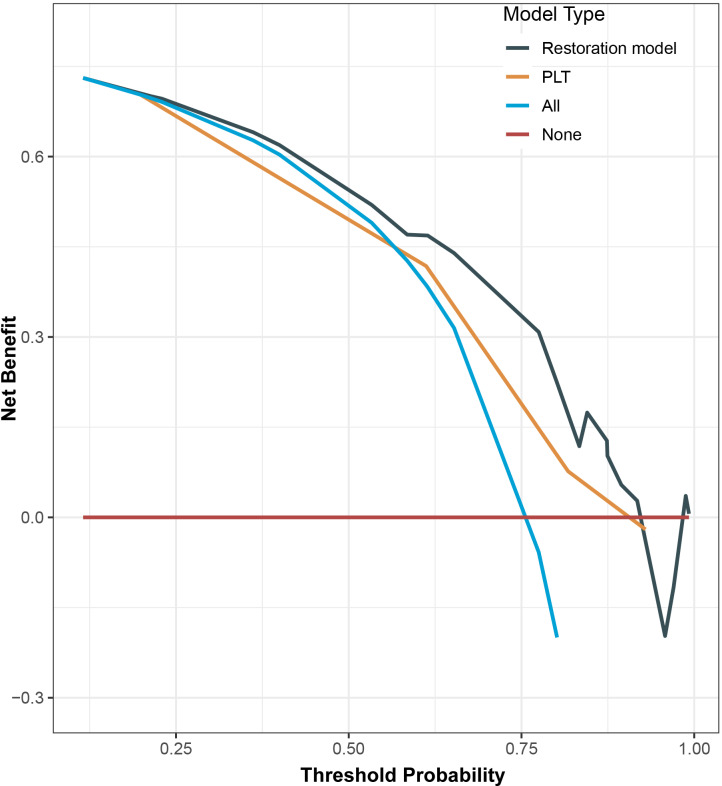
Decision curve analyses showed the clinical benefit of the different indexes. The model-predicted probabilities of 14-day recovery were compared with PLT stratification-predicted probabilities of 14-day recovery. Solid lines indicate the net benefits of the predictive indexes across a range of threshold probabilities (black: Restoration model; orange: PLT stratification). The horizontal solid red line represents the assumption that no patient will experience the event, and the solid blue line indicates the assumption that all patients will experience the event. Abbreviations: PLT: platelet.

A nomogram was used to demonstrate the interrelationships among variables selected by Cox regression analysis (the stratification of PLT, PT, and days from onset to admission) and integrated them onto the same plane. Points were assigned to each value level of each predictor following the degree to which they contributed to the outcome event (i.e., the magnitude of the regression coefficients), among which 0 points were assigned for PLT ≤ 30 × 10^9^/L, PT > 13s, and days from onset to admission >6 days, and 100 points were assigned for PLT > 90 × 10^9^/L. Then, the total points obtained by summing the single points underwent a functional transformation with the probability of the occurrence of the outcome event to calculate the probability of a patient achieving PLT recovery within 14 days. The higher the total points, the greater the probability of platelet recovery within 14 days (Fig 4). The DCA curves for the Cox regression model of PLT recovery and the single PLT stratification predictor were plotted to compare the clinical net benefit of both, we found that the curve of the single PLT predictor model had an intersection with the curve of the all-intervention model, and the clinical net benefit of the single PLT predictor exceeded that of the all-intervention after the intersection. Furthermore, the clinical net benefit of the PLT recovery model all exceeded that of the single PLT stratification predictor across a certain range of threshold probabilities (Fig 5).

## Discussion

In this study, we constructed a Cox proportional hazard model associated with PLT recovery of SFTS patients to explore independent risk factors, which identified early treatment as a decisive factor in platelet recovery, providing clinicians with a practical approach to improve decision-making and avoid adverse clinical events.

Many prior studies have established risk prediction models for SFTS patients using death as the outcome event. Sun J et al. [14] found that a longer time from onset to confirmed diagnosis was correlated with an increased risk of death, and that the interval from onset to confirmed diagnosis was a critical period for treatment, during which prolongation could have led to missed therapy opportunities. Meanwhile, Li H et al. [5] suggested that patients with longer delays before hospital admission (an additional day before admission was associated with an OR of 1.18) had a higher mortality rate. In line with previous studies, we found that days from onset to admission of more than six days was an independent risk factor for platelet recovery within 14 days. Additionally, the Kaplan-Meier curves also confirmed that days from onset to admission of more than six days was detrimental to platelet recovery in SFTS patients. The sixth day of duration of disease is the period in which the fever stage and multiple organ failure stage overlap. The fever stage lasts for 5–11 days, and most patients enter the multiple organ failure stage after approximately 5 days of onset, which lasts for 7–14 days [10]. The viral load in SFTS survivors usually decreases gradually at the multiple organ failure stage, but is still higher in fatal cases of SFTS [20], thereby indicating the significance of shortening the time interval between onset and confirmed diagnosis, followed by early therapeutic intervention, to improve prognosis.

SFTSV has a pan-tropic nature, allowing it to invade various organs in patients and cause organ damage, which in turn leads to elevated laboratory markers [21]. Therefore, changes in SFTS laboratory markers can be used to predict disease progression and prognosis in patients [22,23]. Kato H et al. [24] revealed that baseline PLT was a risk predictor of influencing the mortality rate of SFTS patients, and the lower the PLT, the poorer the prognosis for patients. The PLT of severe SFTS patients declined more obviously than in those with mild SFTS [25]. However, the above studies did not determine the specific risk prediction level associated with baseline PLT values, as they did not develop independent risk analysis or perform stratification. Our study indicated that baseline PLT greater than 90 × 10^9^/L was a favorable predictor of 14-day recovery, and when PLT was 30 × 10^9^/L or less at baseline, there was the lowest recovery rate of SFTS patients observed through Km curves, which verified the importance of early treatment. Previous studies have confirmed that patients in the death group exhibited significant coagulation abnormalities, and that elevated PT was an important risk factor for prolonged SFTS course [26], thus affecting the prognosis of SFTS patients [27,28]. Moreover, Li Z et al. [29] found that activated partial thromboplastin time (APTT) was a predictor of unfavorable outcomes in patients with SFTS, which was inconsistent with this study. Our analysis included PT instead of APTT because there was no significant difference between the two groups, which might be due to the inadequacy of PT as a substitute. Mizoe et al. [30] have demonstrated that coagulation factor XI was the likely cause of APTT prolongation in SFTS, and that plasma-derived or recombinant coagulation factors can be used as alternatives to treat bleeding tendencies to maintain a normal PT if coagulation factor deficiency triggers a prolonged APTT interval [31]. Therefore, if the baseline PT was abnormal, it was suggested that the patient was more severe compared to patients with normal PT, which might demonstrate a higher recovery probability beyond 14 days. Consequently, PT abnormalities may have a higher predictive value in predicting recovery after 14 days than APTT abnormalities.

Currently, there were no specific therapy for SFTS, and and the recovery mainly depended on supportive treatment [32]. Some proposed treatment for SFTS include antiviral drugs, which included two main categories: small molecule drugs (known as favipiravir and ribavirin), macromolecular drugs (Neutralizing antibodies). Recent research had isolated SF5 and SF83 located on the Gn subdomain I, where may be the hot spots in the SFTS therapeutic agents [33]. Moreover, previous study had proposed that favipiravir might be more effective than ribavirin [32], which did not affect platelet counts in patients with fatal or non-fatal disease during the hospital stay [34]. However, the effect of favipiravir was significant only among patients aged ≤70 years, with onset-to-admission interval ≤5 days, therapy duration ≥5 days or baseline viral load ≤ 1 × 10^6^ copies/mL [35]. In our study, although 92.3% patients were used favipiravir and 69% patients were ≤70 years, 47% patients had onset-to-admission interval ≤5 days and 35% patients had therapy duration ≥5 days, thereby indicating the antivirals may have no influence on 14 days PLT recovery and the importance of early application of antiviral drugs to benefit patients. At the same time, we found that supportive therapy was more effective than antivirals in univariate Cox regression model, further emphasizing the importance of early supportive therapy. Previous studies had identified that SFTS occurring acute kidney injury and neurological complications were associated with the 14-day mortality rate [17], and SFTS patients with fungus infections have a higher mortality rate [36], which verified non-recovery group had a higher incidence proportion in our study. Additionally, prolonged high-dose IVIG was beneficial to the prognosis in SFTS patients with neurological complications [12], thereby identifying non-recovery group had a higher application proportion (IVIG, CRRT, antifungal therapy) for the above complications. Moreover, a study had suggested that thrombocytopenia in SFTSV was a multifactor-process involving host immune response and PLT transfusions alone showed minor role in improving prognosis [37], which corroborated that platelet transfusions did not accelerate the platelet recovery rate in our study with no difference between the two groups.

Furthermore, Liu QQ et al. [7] previously constructed a hazard prediction model for the early diagnosis of death in SFTS patients, indicating that age over 60 years was an independent risk factor. However, our results identified that age had no influence on PLT recovery in the multivariate model and was not an independent predictor, which might be correlated with the exclusion of deceased patients from our analysis. This indicated that age was more associated with death, confirming the findings of the prior study. He Z et al. [38] revealed that diarrhea at the time of hospital admission was associated with fatal outcomes. Similarly, our univariate Cox regression analysis found that diarrhea was a risk factor for platelet recovery. Therefore, clinicians should promptly implement appropriate treatment measures for SFTS patients exhibiting diarrhea symptoms at admission to prevent the disease worsening.

Finally, we performed a nomogram to visualize risk predictors, providing a convenient method of predicting recovery rate for clinicians. The Cox regression model had an AUC of 0.745 in our study, which had good predictive power. Moreover, the DCA curves demonstrated that as the probability of recovery exceeded approximately 50%, the clinical net benefit from focusing solely on the PLT factor model began to consistently surpass that from the all-intervention model, and the distance between the two models increased. This finding emphasized the critical importance of initiating early treatment when there was no significant decrease in baseline PLT, to achieve greater clinical net benefits. All in all, our study has several advantages: firstly, it was the first time to analyze PLT recovery using 14 days as the time cutoff value. Secondly, for the first time, we defined PLT recovery as an outcome event, and stratified PLT, PT, and days from onset to admission to clarify the critical values of risk factors. Thirdly, the variables screened in the Cox regression model were objective indicators, and there were no subjective indicators requiring human judgment, which made it easier to validate as well as simpler and more convenient to use in clinical diagnosis and treatment. Our study laid a theoretical basis to guide primary care clinicians in providing early detection, diagnosis, and treatment of patients and proposed that patients could improve more quickly if they sought timely medical attention within six days of onset. Physicians should pay attention to changes in both PLT and PT at the time of admission to identify patients with poor prognoses early to improve patient survival.

Our research also has several limitations. First, this study was a single-center retrospective study and lacked internal and external validation, resulting in limited generalizability of the findings. Therefore, its conclusion should be further validated in multiple centers. Second, Previous studies have shown that high viral load at baseline was associated with disease prognosis [39,40] and that a nomogram model incorporating viral load had better predictive accuracy [41], but due to the limited cost of quantitative SFTS RNA testing in the region where the experiment was conducted, which led to an excessive number of missing values for disease load at baseline, this variable could not be included in the study. Third, co-infection with other tick-borne pathogens could not be excluded.

## Conclusion

SFTS patients with baseline platelet count greater than 90 × 10^9^/L, days from onset to admission of less than 6 days, and baseline PT of less than 13 seconds may have their PLT recovered rapidly during convalescence. This model demonstrates good predictive capability and clinical benefit, aiding clinicians in advocating for early treatment, early identification of patients with poor prognosis, and better management of the disease.

## Supporting information

S1 Table Raw data on baseline clinical symptoms, baseline laboratory indicators, treatments, comorbidities in SFTS patients. (XLSX)

## References

[1] Diagnosis and treatment protocol for fever with thrombocytopenia syndrome (2023 edition). Chin J Infect Control. 2024;23(7):918–20. doi: 10.12138/j.issn.1671-9638.20245430

[2] Park SY, Kwon J-S, Kim JY, Kim S-M, Jang YR, Kim M-C, et al. Severe fever with thrombocytopenia syndrome-associated encephalopathy/encephalitis. Clin Microbiol Infect. 2018;24(4):432.e1–432.e4. doi: 10.1016/j.cmi.2017.09.002 28899841

[3] Fang L, Yu S, Tian X, Fu W, Su L, Chen Z, et al. Severe fever with thrombocytopenia syndrome virus replicates in platelets and enhances platelet activation. J Thromb Haemost. 2023;21(5):1336–51. doi: 10.1016/j.jtha.2023.02.006 36792011

[4] Li X-K, Lu Q-B, Chen W-W, Xu W, Liu R, Zhang S-F, et al. Arginine deficiency is involved in thrombocytopenia and immunosuppression in severe fever with thrombocytopenia syndrome. Sci Transl Med. 2018;10(459):eaat4162. doi: 10.1126/scitranslmed.aat4162 30232226

[5] Li H, Lu Q-B, Xing B, Zhang S-F, Liu K, Du J, et al. Epidemiological and clinical features of laboratory-diagnosed severe fever with thrombocytopenia syndrome in China, 2011-17: a prospective observational study. Lancet Infect Dis. 2018;18(10):1127–37. doi: 10.1016/S1473-3099(18)30293-7 30054190

[6] Yu CZ, Ayinuer T, Dilinuer W, Lei Q, Liu W. Construction of a predictive model for mortality risk in fever with thrombocytopenia syndrome. Chin J Clin Infect Dis. 2023;16(5):354–9. doi: 10.3760/cma.j.issn.1674-2397.2023.05.003

[7] Liu QQ, Zhang ZR. Establishment of a mortality prediction model for fever with thrombocytopenia syndrome. Med Inf. 2023;36(11):21–6. doi: 10.3969/j.issn.1006-1959.2023.11.004

[8] Chen ZQ, Pei SJ, Suo WS, Wang XY, Li Y, Huang XY. Research progress on risk factors for mortality in patients with fever with thrombocytopenia syndrome. Int J Virol. 2023;30(5):432–6. doi: 10.3760/cma.j.issn.1673-4092.2023.05.019

[9] Chen QL, Zhu MT, Chen N, Yang D, Yin WW, Mu D, et al. Epidemiological characteristics of severe fever with thtrombocytopenia syndrome in China, 2011-2021. Zhonghua Liu Xing Bing Xue Za Zhi. 2022;43(6):852–9. doi: 10.3760/cma.j.cn112338-20220325-00228 35725341

[10] Gai Z-T, Zhang Y, Liang M-F, Jin C, Zhang S, Zhu C-B, et al. Clinical progress and risk factors for death in severe fever with thrombocytopenia syndrome patients. J Infect Dis. 2012;206(7):1095–102. doi: 10.1093/infdis/jis472 22850122

[11] Liu JY, Feng J, Li AL, Wang SY, Zheng R, Chen HZ. Clinical diagnosis, treatment, and analysis of risk factors for mortality in patients with bunyavirus infection associated with fever and thrombocytopenia syndrome. Chin J Med Res. 2018;41(5):429–33. doi: 10.3760/cma.j.issn.1673-4904.2018.05.012

[12] Liu Y, Tong H, He F, Zhai Y, Wu C, Wang J, et al. Effect of intravenous immunoglobulin therapy on the prognosis of patients with severe fever with thrombocytopenia syndrome and neurological complications. Front Immunol. 2023;14:1118039. doi: 10.3389/fimmu.2023.1118039 37033957 PMC10073413

[13] Park S-W, Lee C-S, Kim J-H, Bae I-G, Moon C, Kwak YG, et al. Severe fever with thrombocytopenia syndrome: comparison with scrub typhus and clinical diagnostic prediction. BMC Infect Dis. 2019;19(1):174. doi: 10.1186/s12879-019-3773-1 30782137 PMC6381645

[14] Sun J, Gong Z, Ling F, Zhang R, Tong Z, Chang Y, et al. Factors associated with severe fever with thrombocytopenia syndrome infection and fatal outcome. Sci Rep. 2016;6:33175. doi: 10.1038/srep33175 27605309 PMC5015071

[15] Zhao D, Xu W, Zhan Y, Xu L, Ding W, Xu A, et al. Development and validation of nomograms to predict the prognosis of patients with unresectable hepatocellular carcinoma receiving transarterial chemoembolization. Clin Med Insights Oncol. 2023;17:11795549231178178. doi: 10.1177/11795549231178178 37378393 PMC10291869

[16] Shang H, Wang LL, Yin YB. Experimental diagnostics. Beijing: People’s Health Publishing House; 2015 May.

[17] Xiong L, Xu L, Lv X, Zheng X. Effects of corticosteroid treatment in patients with severe fever with thrombocytopenia syndrome: a single-center retrospective cohort study. Int J Infect Dis. 2022;122:1026–33. doi: 10.1016/j.ijid.2022.07.001 35803466

[18] Fang K, Song X, Bo J. A nomogram to predict mortality in patients with severe fever with thrombocytopenia syndrome. Sci Rep. 2024;14(1):10627. doi: 10.1038/s41598-024-60923-9 38724615 PMC11081946

[19] Miao D, Liu M-J, Wang Y-X, Ren X, Lu Q-B, Zhao G-P, et al. Epidemiology and ecology of severe fever with thrombocytopenia syndrome in China, 2010‒2018. Clin Infect Dis. 2021;73(11):e3851–8. doi: 10.1093/cid/ciaa1561 33068430 PMC8664468

[20] Cui N, Bao X-L, Yang Z-D, Lu Q-B, Hu C-Y, Wang L-Y, et al. Clinical progression and predictors of death in patients with severe fever with thrombocytopenia syndrome in China. J Clin Virol. 2014;59(1):12–7. doi: 10.1016/j.jcv.2013.10.024 24257109

[21] Li S, Li Y, Wang Q, Yu X, Liu M, Xie H, et al. Multiple organ involvement in severe fever with thrombocytopenia syndrome: an immunohistochemical finding in a fatal case. Virol J. 2018;15(1):97. doi: 10.1186/s12985-018-1006-7 29848330 PMC5977472

[22] Jia B, Yan X, Chen Y, Wang G, Liu Y, Xu B, et al. A scoring model for predicting prognosis of patients with severe fever with thrombocytopenia syndrome. PLoS Negl Trop Dis. 2017;11(9):e0005909. doi: 10.1371/journal.pntd.0005909 28934195 PMC5626493

[23] Shin J, Kwon D, Youn S-K, Park J-H. Characteristics and factors associated with death among patients hospitalized for severe fever with thrombocytopenia syndrome, South Korea, 2013. Emerg Infect Dis. 2015;21(10):1704–10. doi: 10.3201/eid2110.141928 26402575 PMC4593431

[24] Kato H, Yamagishi T, Shimada T, Matsui T, Shimojima M, Saijo M, et al. Epidemiological and clinical features of severe fever with thrombocytopenia syndrome in Japan, 2013-2014. PLoS One. 2016;11(10):e0165207. doi: 10.1371/journal.pone.0165207 27776187 PMC5077122

[25] Li W. Epidemiological and clinical characteristics analysis of fever with thrombocytopenia syndrome. Chin J Pathogen Biol. 2017;12(9):901–4. doi: 10.13350/j.cjpb.170923

[26] Zhang Y, Zhong P, Wang L, Zhang Y, Li N, Li Y, et al. Development and validation of a clinical risk score to predict the occurrence of critical illness in hospitalized patients with SFTS. J Infect Public Health. 2023;16(3):393–8. doi: 10.1016/j.jiph.2023.01.007 36706468

[27] Hou HH, Mao LL, Liang YH, Liu Y, Liu XS, Deng BC. Clinical characteristics and prognostic factors of fever with thrombocytopenia syndrome in Dalian, Liaoning Province. Chin J Infect Control. 2021;20(10):897–902. doi: 10.12138/j.issn.1671-9638.20218284

[28] Han CX, Sun AJ, Pu CW, Li YT, Sui F, Qin SJ. Epidemiological characteristics and prognostic indicators of fever with thrombocytopenia syndrome caused by novel bunyavirus infection. Chin J Hosp Infect. 2019;29(2):171–4+187. doi: 10.11816/cn.ni.2019-180685

[29] Li Z, Zhang Z, Chen C. Novel nomograms to predict risk and prognosis in hospitalized patients with severe fever with thrombocytopenia syndrome. Front Med (Lausanne). 2023;10:1321490. doi: 10.3389/fmed.2023.1321490 38105896 PMC10722171

[30] Mizoe A, Sakaue J, Takahara N. Why does activated partial thromboplastin time prolongation occur in severe fever with thrombocytopenia syndrome? BMJ Case Rep. 2020;13(10):e235447. doi: 10.1136/bcr-2020-235447 33033003 PMC7545498

[31] Peters R, Harris T. Advances and innovations in haemophilia treatment. Nat Rev Drug Discov. 2018;17(7):493–508. doi: 10.1038/nrd.2018.70 29880919

[32] Zhang Y, Huang Y, Xu Y. Antiviral treatment options for severe fever with thrombocytopenia syndrome infections. Infect Dis Ther. 2022;11(5):1805–19. doi: 10.1007/s40121-022-00693-x 36136218 PMC9510271

[33] Chang Z, Gao D, Liao L, Sun J, Zhang G, Zhang X, et al. Bispecific antibodies targeting two glycoproteins on SFTSV exhibit synergistic neutralization and protection in a mouse model. Proc Natl Acad Sci U S A. 2024;121(24):e2400163121. doi: 10.1073/pnas.2400163121 38830098 PMC11181109

[34] Shimojima M, Fukushi S, Tani H, Yoshikawa T, Fukuma A, Taniguchi S, et al. Effects of ribavirin on severe fever with thrombocytopenia syndrome virus in vitro. Jpn J Infect Dis. 2014;67(6):423–7. doi: 10.7883/yoken.67.423 25410555

[35] Yuan Y, Lu Q-B, Yao W-S, Zhao J, Zhang X-A, Cui N, et al. Clinical efficacy and safety evaluation of favipiravir in treating patients with severe fever with thrombocytopenia syndrome. EBioMedicine. 2021;72:103591. doi: 10.1016/j.ebiom.2021.103591 34563924 PMC8479638

[36] Song H, Zou S, Huang Y, Wang Y, Wang T, Wei W, et al. The pathogenic and clinical characteristics of severe fever with thrombocytopenia syndrome patients with co-infections. Front Cell Infect Microbiol. 2023;13:1298050. doi: 10.3389/fcimb.2023.1298050 38106473 PMC10722497

[37] Li X-K, Dai K, Yang Z-D, Yuan C, Cui N, Zhang S-F, et al. Correlation between thrombocytopenia and host response in severe fever with thrombocytopenia syndrome. PLoS Negl Trop Dis. 2020;14(10):e0008801. doi: 10.1371/journal.pntd.0008801 33119592 PMC7595704

[38] He Z, Wang B, Li Y, Du Y, Ma H, Li X, et al. Severe fever with thrombocytopenia syndrome: a systematic review and meta-analysis of epidemiology, clinical signs, routine laboratory diagnosis, risk factors, and outcomes. BMC Infect Dis. 2020;20(1):575. doi: 10.1186/s12879-020-05303-0 32758175 PMC7409422

[39] Wang D, Cao K, Shen X, Zhang B, Chen M, Yu W. Clinical characteristics and immune status of patients with severe fever with thrombocytopenia syndrome. Viral Immunol. 2022. doi: 10.1089/vim.2021.0217 35675657

[40] Li JE, Niu TH. Correlation between early laboratory indicators and prognosis of fever with thrombocytopenia syndrome. Anhui Med J. 2019;40(4):426–9. doi: 10.3969/j.issn.1000-0399.2019.04.021

[41] Xiong S, Zhang W, Li M, Xiong Y, Li M, Wang H, et al. A simple and practical score model for predicting the mortality of severe fever with thrombocytopenia syndrome patients. Medicine (Baltimore). 2016;95(52):e5708. doi: 10.1097/MD.0000000000005708 28033271 PMC5207567

